# Comparison of the Efficacy of Human Umbilical Cord-Derived and Bone Marrow Aspirate Concentrate Mesenchymal Stem Cells for Cartilage Repair Defects of the Knee via Arthroscopic Implementation on Scaffolds in a Retrospective Study

**DOI:** 10.3390/jpm14030220

**Published:** 2024-02-20

**Authors:** Karol Pałka, Bogusław Sadlik, Paweł Kamiński, Rafał J. Bułdak, Michał Dobrakowski

**Affiliations:** 1 Department of Biochemistry, Faculty of Medical Sciences in Zabrze, Medical University of Silesia in Katowice, Jordana 19, 41-808 Zabrze, Poland; 2 College of Physiotherapy in Wrocław, ul. Kościuszki 4, 50-038 Wrocław, Poland; 3 Department of Radiology and Radiodiagnostics, Public Clinical Hospital, Medical University of Silesia in Katowice, 3-go Maja 13-15, 41-800 Zabrze, Poland; 4 Department of Clinical Biochemistry and Laboratory Diagnostics, Institute of Medical Sciences, University of Opole, Oleska 48, 45-052 Opole, Poland

**Keywords:** cartilage, knee, regenerative medicine, mesenchymal stem cells

## Abstract

Injuries to the articular cartilage of the human knee joint, commonly resulting from trauma, inflammation, or age- and activity-related wear and tear, have significant implications, primarily leading to osteoarthritis (OA). Conservative treatments for such injuries often yield suboptimal clinical outcomes. Surgical interventions using current methods may not consistently provide satisfactory results, largely due to the formation of low-quality scar tissue lacking the biomechanical properties of hyaline cartilage. In this retrospective study, we compared the results of two promising methods for regenerating cartilage defects in the knee joints using scaffolds soaked with stem cells of different origins: bone marrow aspirate concentrate mesenchymal stem cells (BMAC-MSCs) and human umbilical cord-derived mesenchymal stem cells (hUCB-MSCs). We evaluated 39 patients (39 knees, hUCB-MSCs: 20 knees, BMAC: 19 knees) at the 12-month follow-up using VAS, KOOS, Lysholm scales and radiologically with M-MOCART 2.0 score. The analysis demonstrated substantial overall improvement in both groups, notably reflected in enhanced quality of life for the patients. Interestingly, the final scores in the hUCB-MSCs group were comparable to those in the BMAC-MSCs group, with no statistically significant differences observed, despite variations in preoperative age and lesion size. Notably, the hUCB-MSCs group consisted of significantly older individuals with larger lesion sizes. Both procedures were found to be safe, and improvement was observed in both groups, which holds promise for future clinical investigations.

## 1. Introduction

Osteoarthritis (OA) poses a significant challenge to the health of elderly adults, as it is one of the leading causes of disability. The burden of this disease is expected to worsen due to the increasing prevalence of obesity and the aging of the population [1]. OA is a widespread condition affecting over 500 million people worldwide, representing approximately 6% of the global population. The incidence of this disease has been on the rise, with a 48% increase reported between 1990 and 2019 [2]. The damage to the articular cartilage often leads to gradual wear and tear, which may cause osteoarthritic alterations in the joint, leading to increased pain and reduced function.

Cartilage lesions have limited ability to heal on their own, which has prompted researchers to explore novel treatment strategies aimed at enhancing cartilage regeneration [3]. In the advanced stages of OA, arthroplasty, which involves replacing the affected joint with a prosthetic device, is the most commonly used treatment option. However, the prosthetic device has a finite lifespan and with the aging population, there has been a steady increase in the number of arthroplasties that require revision and replacement. These secondary procedures are more complicated, costly, and less successful than the initial surgery, creating a significant challenge for healthcare systems [4].

A biological approach at the earlier stage of OA has the potential to offer a more durable and long-lasting solution [5]. Currently, there are several surgical options accessible for managing and treatment of local cartilage damage. Microfractures (MFx), osteochondral allografts and autografts implantations (OAT), cell-free implants, autologous chondrocyte implantation (ACI), and finally matrix-based chondrogenesis [6]. Regarding the current recommendations and guidelines for selecting appropriate cartilage repair methods, the primary factor in decision-making remains the size of the defect. Each of the previously mentioned methods has its advantages and drawbacks. Limitations for the methods are as follows: MFx alone leads to poor quality of the tissue, which can lead to inferior long-term results [7]. Limitations of OAT may arise from factors such as donor site availability and morbidity, as well as technical challenges that can restrict treatment to smaller lesions, typically measuring less than 4 cm^2^ [8]. Cell-free implants alone do not support the biological impact on the affected chondral lesion in the knee. Matrix-guided autologous chondrocyte transplantation is a costly (ten times more expensive than MFx), two-step procedure that is still evolving and has some limitations in worldwide approvals [9].

In recent years, a one-stage surgical technique called autologous matrix-induced chondrogenesis (AMIC) has emerged, aiming to leverage the benefits of previously mentioned procedures. This approach involves the use of a matrix that provides three-dimensional growth conditions for the cells [10], stimulating the differentiation of progenitor cells into chondrocytes, while also shielding the cells from mechanical overload [11]. In the latest meta-analysis, the AMIC procedure for focal chondral lesions has been superior to MFX, OAT, or ACI in terms of rate of failure and revisions in 3-year follow-up [12].

Mesenchymal stem cells (MSCs) used for chondrogenesis in AMIC procedures can be obtained from various sources, such as bone marrow (BM), adipose tissue (AT), placenta, and umbilical cords (UC) [13] and the effectiveness of MSCs therapy in alleviating pain and improving clinical symptoms associated with osteoarthritis (OA) has been demonstrated [14,15]. MSCs have the capacity for self-renewal and differentiation into various cell types. They showcase therapeutic promise in treating OA through specific differentiation, immune response modulation, anti-inflammatory activity, support for angiogenesis, enhancement of the local environment, and promotion of tissue regeneration [16].

In recent studies, human umbilical cord-derived mesenchymal stem cells (hUCB-MSCs) might be the most effective for improving function among other MSCs in knee osteoarthritis injection treatment [17].

To the best of our knowledge, there are only a few published research studies comparing the efficacy of different stem cell therapies in surgical cartilage repair. Taking into account the aforementioned considerations, our study is focused on integrating the most promising surgical technique (AMIC) with the most favorable MSC source (hUCB-MSCs) and conducting a comparative analysis with the cost-effective MSC sources using bone marrow aspirated concentrate mesenchymal stem cells (BMAC-MSCs). First attempts of research in the direction of comparison collagen matrices induced by hUCB-MSCs and BMAC-MSCs were made in the last few years [18,19,20]. Ryu et al. compared 25 BMAC-MSCs patients versus 27 hUCB-MSCs patients achieving comparable results in both groups after a 2-year follow-up [18]. Yang et al. compared 55 patients in each group with an additional procedure of high tibial osteotomy (HTO) attaining similar outcomes within both groups after a mean follow-up of 33 months [19]. Lee et al. compared the BMAC group composed of 42 cases and the hUCB-MSCs group composed of 32 cases (with additional HTO in both groups) obtaining a better outcome in the hUCB-MSCs group with a minimum of 1 year of follow-up [20].

To the best of our current understanding, this is the first study using hUCB-MSCs knee implantation outside South Korea where an officially registered product was used (Cartistem^®^). Thus, our study seeks to investigate which cell therapy approach between those two (BMAC-MSCs and hUCB-MSCs) is more effective for the one-step cartilage repair surgical procedure and compare results with the previous ones without the necessity of using a specific product (Cartistem^®^), which can lead to increased accessibility and reproducibility. Our study aims to provide valuable insights and contribute to the ongoing efforts to optimize stem cell therapies for surgical cartilage repair, ultimately improving patient outcomes and quality of life.

## 2. Materials and Methods

A retrospective review was conducted on the patients who underwent cartilage repair surgery using AMIC technique with BMAC and hUCB-MSCs between 2015 and 2019. Two of the co-authors performed all the surgeries by themselves. To conduct this study, the following inclusion criteria were established: (1) 15–70 years old, with a body mass index (BMI) < 35 kg/m^2^, (2) Kellgren–Lawrence (K–L) severity grade I, II or III in the index knee for radiographic classification of OA, (3) full-thickness cartilage defect at least 2 cm^2^ in MRI scans, (4) a stable knee joint or sufficiently reconstructed ligaments. If not, ligament repair must be completed before, during, or within 6 weeks after cartilage treatment, (5) free range of motion of the affected knee joint or ≤10° of extension and flexion loss, (6) visual analog score(VAS)of pain > 3, (7) ability to give written informed consent to participate in the study and to comply with all study requirements, including attending all follow-up visits and assessments and postoperative rehabilitation regimen.

Exclusion criteria were as follows: (1) active cancer or cancer reported in a medical history of which treatment ended less than 5 years ago, (2) allergy to antibiotics used in cell culture, (3) any other disease or condition that may change the accuracy of test results (including rheumatoid arthritis, endocrine diseases such as diabetes, autoimmune diseases, thyroid disease, connective tissue diseases such as collagenosis, osteoporosis, chronic renal failure, Lyme disease, (4) corticosteroids or cytotoxic, immunosuppressive or immunomodulating drugs intake in the last 30 days before surgery, (5) known history of HIV, HBV, or HCV infection (positive laboratory test), (6) subject is a woman who is pregnant or breastfeeding at the screening visit or a woman of child bearing potential who refuses to use effective contraception during the course of study, (7) contraindication to performing MRI examination, (8) within last 6 months involved in any other research study involving taking drugs which could have influence to outcome of current study trial.

A total of 39 patients were included in the study (39 knees, hUCB-MSCs: 20 knees, BMAC: 19 knees). All subjects gave their informed consent for inclusion before they participated in the study. The study was conducted in accordance with the Declaration of Helsinki and the protocol was approved by the Ethics Committee of Beskidzka Izba Lekarska (2015/06/25/1/BIL).

### 2.1. Cell Culture and Preparation of hUCB-MSCs Isolate

The collected samples of umbilical cords after obtaining informed consent from the donor are kept in a temperature-controlled environment and processed within 48 h of procurement. The umbilical cord segment is first washed in a sterile solution of saline and antibiotic–antimycotic fluid and then divided into 2 cm long pieces while removing blood vessels to isolate the Wharton’s jelly. The Wharton’s jelly is then sectioned into 2 cm^3^ fragments, which are cultured in a xeno-free medium supplemented with antibiotics for 2 to 3 weeks at 37 °C. Once the stem cells reach 90% confluence, they are collected and the remaining tissue is discarded. The Wharton’s jelly mesenchymal stem cells (WJ-MSCs) are then reseeded for further expansion. To ensure the viability of the WJ-MSCs, trypan blue exclusion in a hemocytometer is used to verify the expanded cell lines, and immunophenotyping is conducted to confirm the presence of surface markers CD73, CD90, and CD105, as well as the absence of markers CD14, CD19, CD34, CD45, and HLA DR. Finally, the isolated WJ-MSCs are placed into freezing bags and suspended in a mixture containing human albumin and 10% dimethyl sulfoxide before being cooled in a controlled-rate freezer and stored at a temperature of −195 °C in liquid nitrogen. The whole procedure is made by the Polish Stem Cell Bank and has been approved by ethical review board.

### 2.2. hUCB-MSCs-Embedded Scaffold Preparation, BMAC-MSCs-Embedded Scaffold Preparation, and Dry Arthroscopic Implantation

The WJ-MSC suspension is thawed from liquid nitrogen storage and warmed in a water bath at 37 °C. A 2-step centrifugation process is used to remove dimethyl sulfoxide from the WJ-MSC suspension. The cells are resuspended in saline solution and centrifuged twice, then the pellet of cells is suspended in 1 mL of saline solution and transferred to a sterile syringe for implantation. During knee arthroscopy, the patient is placed under anesthesia and a complete assessment of the articular cartilage is performed to confirm the location and size of the cartilage lesion. The same steps are taken for both BMAC-MSCs and hUCB-MSCs groups during the surgery procedure in addition to the BMAC preparation, which requires BM extraction from the proximal tibia bone using 20 mL syringe containing 2 mL of the anticoagulant. The collected material was subjected to centrifugation at 2500 rpm for 5 min to concentrate the BM cells. The concentrated BM was then carefully collected using a fresh heparinized syringe, which was anticipated to contain the accumulated MSCs [21]. In next stages of the knee arthroscopy, loose cartilage is removed, and the cartilage lesion is carefully prepared with vertical peripheral walls to the depth of subchondral bone using specialized arthroscopic instruments if necessary. This preparation is a crucial component of the procedure that will optimize regeneration of articular cartilage. After accurate sizing of the cartilage defect using an arthroscopic measuring device, a template of the lesion is created on a sterile table using a sterile latex dental dam. The template is then applied to the defect multiple times to ensure accuracy and resized as necessary. In our study, we used three different scaffolds—Hyalofast^®^, Chondrogide^®^, and Biomerg^®^. A size-matched scaffold is created and moistened with saline solution immersed in a WJ-MSC suspension and adequately for BMAC-MSCs group in BMAC solution (both solutions prepared and described as previously outlined), for 5 min to create the final embedded scaffold graft. The prepared defect is then confirmed with thorough arthroscopic visualization, and the joint space is drained to prepare for graft implantation. A specialized retraction system may be used to optimize access to the defect, and a valveless cannula or skid is placed into the working portal to equalize pressure and prevent collapse of soft tissues. The embedded scaffold is then introduced into the knee compartment through the skid or valveless cannula and placed into the defect using a specialized graft-inserting instrument. After gentle seating and securing of the graft, fibrin glue is applied around the periphery to improve fixation. The knee is then cycled through flexion and extension to confirm secure position of the implant within the prepared defect. Finally, the surgical incisions are closed appropriately, a sterile dressing is applied, and a brace is positioned to immobilize the knee in extension. The details of procedures with their advantages, limitations, pearls, and pitfalls are well described in our previous work [22].

### 2.3. Clinical Evaluation and Magnetic Resonance Evaluation

We utilized the Lysholm scale [23], the Knee Injury and Osteoarthritis Outcome Score (KOOS) [24], and the Visual Analogue Scale (VAS) pain score (the higher the result, the worse the pain) to evaluate the pain and functional disability of the knee before surgery, as well as 6 months and 1-year post-surgery. The modified Magnetic Resonance Observation of Cartilage Repair Tissue (M-MOCART) score in version 2.0 [25] was used to analyze the MRI images, which were taken up to 6 months post-op and more than 12 months. The M-MOCART 2.0 score (the higher the result, the better articular cartilage condition) is highly correlated with clinical outcomes [26] and presents better results than M-MOCART in previous version [27]. To prevent any potential bias, the MRI images were evaluated by an independent radiologist with expertise in musculoskeletal imaging. The radiologist was not involved in the patients’ care and was unaware of the study’s objectives to ensure the objectivity of the evaluation.

### 2.4. Rehabilitation Protocol

The essence of the rehabilitation program after reconstruction of the cartilage surface defects is to maintain and restore joint function while providing safe conditions for the remodeling of the biological graft. All patients were involved in the same post-operative protocol. Following surgery, the knee is stabilized in a brace for 5 days and passive motion is applied until 90° of flexion is achieved. Weight-bearing is gradually increased over a period of 6 weeks, starting with restricted weight bearing for the first 4 weeks, followed by partial weight bearing for 2 weeks, and finally, unrestricted weight bearing. Rehabilitation at this stage includes exercises to improve proprioception, muscle conditioning, and restoration of normal gait. Aerobic and strength training begin at 3 months, and MRI is used to monitor the progress of the repair between 3 and 6 months. Sport-specific training may begin at 6–8 months post-surgery, and the patient returns to full sports between 8 and 12 months depending on their progress and the type of sports.

### 2.5. Statistical Analysis

The statistical techniques used for analysis included descriptive statistics, chi-squared test for independence, independent Student’s *t*-test, and Mann–Whitney U test to compare the two groups using SPSS Version 25. Moreover, repeated measures analysis of variance was employed to investigate differences between pain and knee joint assessment measurements in the groups, and dependent Student’s *t*-test was used to compare the two groups on these measures. We have conducted repeated measures of ANOVA separately in each group. To assess the statistical power of the conducted analyses, calculations were performed using GPower 3.1.9.2. The results indicated that in each case, for repeated measures ANOVA, the test power was 1 (100%). However, in the case of pairwise comparisons using *t*-tests, the power was weaker, reaching 90% only for strong effects. The M-MOCART scale was also utilized and analyzed using both repeated measures analysis of variance and dependent Student’s *t*-test to compare the groups over time, and independent Student’s *t*-test was used to compare the groups on the MOCART scale. The statistical significance level was set at the classical threshold of α = 0.05.

## 3. Results

There was no statistically significant difference found between gender groups. The hUCB-MSCs group was significantly older than the BMAC-MSCs group. No differences were observed in the BMI of the participants. At the same time, larger lesion areas were observed in the hUCB-MSCs group than in the BMAC-MSCs group (Table 1).

It was examined whether there were statistically significant differences between the pre-surgery, 6-month, and 1-year Visual Analog Scale (VAS) pain measurements. These analyses were conducted separately in the BMAC-MSCs group and the hUCB-MSCs group. A repeated measures analysis of variance was performed, with pain as the dependent variable and time intervals after the medical intervention as the independent variable. The analysis revealed a statistically significant result in both groups and showed that pain decreased systematically after each measurement. Such differences were observed in both groups (Table 2).

**Figure 1:**
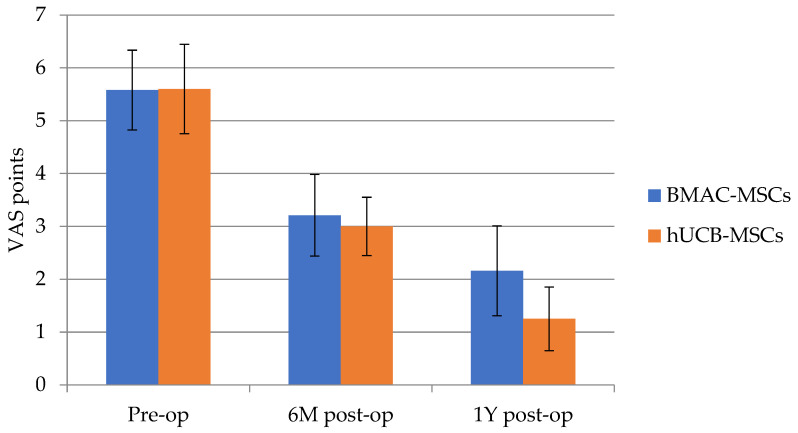
Pre-surgery (pre-op) *p* = 0.971, 6 months (6 M post-op) *p* = 0.664, and 1 year after the surgery (1 Y post-op) *p* = 0.104 VAS pain measurements. There was no significant difference between two groups. BMAC-MSCs (*n* = 20), hUCB-MSCs (*n* = 19).

Next, an independent Student’s *t*-test was performed to compare the BMAC-MSCs group with the hUCB-MSCs group in terms of pain measurements. The test results are not statistically significant, indicating that no significant difference was found between the two groups (Figure 1).

**Figure 2:**
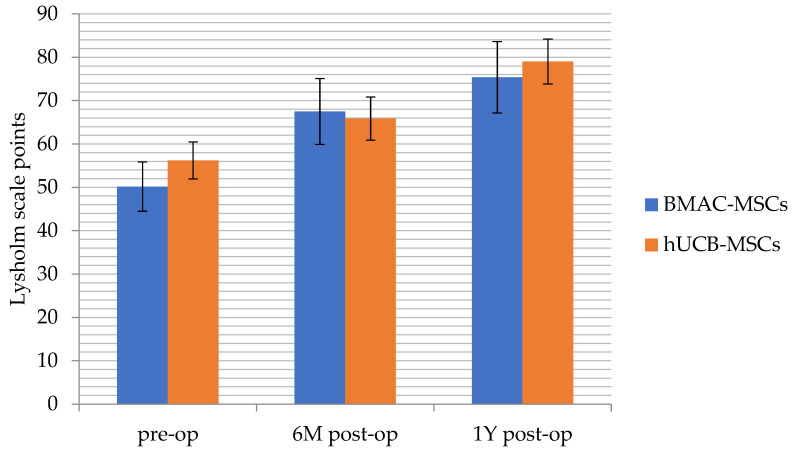
Pre-surgery (pre-op) *p* = 0.102, 6 months (6 M post-op) *p* = 0.726, and 1 year after the surgery (1 Y post-op) *p* = 0.471 Lysholm scale functionality of the knee measurements. As described in the text, both groups progressively improved from measurement to measurement and there were no statistically significant differences between groups. BMAC-MSCs (*n* = 20), hUCB-MSCs (*n* = 19).

Similar comparisons were then performed to determine if there was an improvement in knee joint functionality. Again, a repeated measures analysis of variance was performed, but the dependent variable was the assessment of the knee joint (measured by both the Lysholm and KOOS scales). The results showed no significant differences between the hUCB-MSCs and the BMAC-MSCs group in terms of knee joint assessment according to the Lysholm scale (Figure 2).

The functionality of the knee joint in both groups progressively improved from measurement to measurement, and these differences were statistically significant (Table 3).

The differences in knee joint functionality were then tested using the KOOS scale. The KOOS is a 42-item self-administered questionnaire designed to be self-explanatory, encompassing five patient-relevant dimensions: Other Disease-Specific Symptoms, (KOOS S) Pain (KOOS P), ADL (activities of daily living) Function (KOOS A), Sport and Recreation Function (KOOS SP), and knee-related Quality of Life (KOOS Q). The analysis showed statistically significant effects for each dimension of the functional scale. Almost all pairwise comparisons between measurements were statistically significant. Only in the hUCB-MSCs group, there were no differences between the initial and final measurements for the pain scale and sports and recreational activity. Moreover, in the hUCB-MSCs, the initial measurement for the activities of daily living scale did not differ from the middle measurement. For the remaining comparisons, the following differences were noted: with each measurement, there was a significant improvement in knee joint function, considering all aspects of functionality (Table 4).

From the conducted test, it was shown that the BMAC-MSCs group differed from the hUCB-MSCs group in the initial measurement of the scale of daily living activities and sports and recreational activities. The BMAC-MSCs group had better functioning in the areas of sports and quality of life than the hUCB-MSCs group, while the hUCB-MSCs group had better functioning in daily living activities than the BMAC-MSCs group.

**Figure 3:**
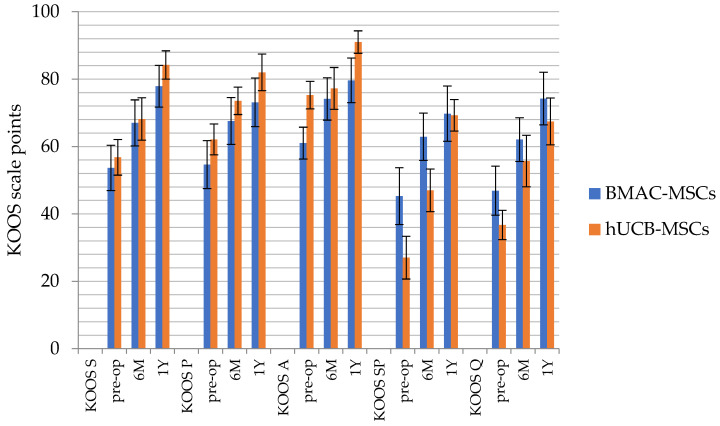
Pre-surgery (pre-op), 6 months (6 M post-op), and 1 year after the surgery (1 Y post-op) KOOS scale functionality of the knee measurements. Other Disease-Specific Symptoms (KOOS S), Pain (KOOS P), ADL (activities of daily living) Function (KOOS A), Sport and Recreation Function (KOOS SP), and knee-related Quality of Life (KOOS Q). BMAC-MSCs (*n* = 20), hUCB-MSCs (*n* = 19).

For the final measurement, statistically significant differences were not observed for most of the scales, but the differences for the scale of daily living activities remained significant. Better functioning in ADL was still observed in the hUCB-MSCs group compared to the BMAC-MSCs group (Figure 3).

To examine the effect of the matrix manufacturer on improvement outcomes, additional comparative analyses were conducted. A mixeddesign 3 × 3 ANOVA was performed. The within-subject factor was the triple measurement of parameters (preoperative measurement vs. measurement 6 months postoperatively vs. measurement 12 months postoperatively), while the between-subject factor was the matrices (Biomerg^®^ vs. Hyalofast^®^ vs. Chondrogide^®^). ANOVA analyses did not reveal a significant interaction effect or statistically significant differences between the matrices.

In the next part of the statistical analysis, descriptive statistics were calculated again, and distributions were checked for the M-MOCART radiological scale (Table 5).

**Figure 4:**
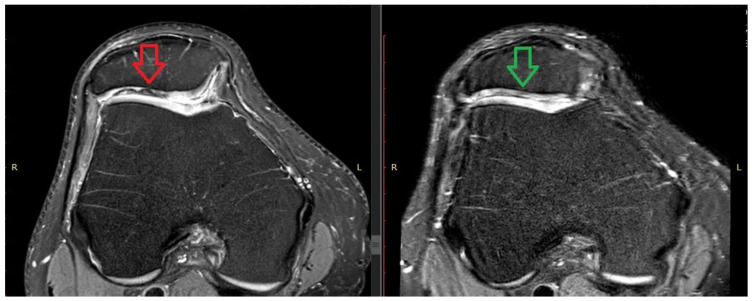
The pre-operation (on the **left**) versus 12 months post-operation (on the **right**) MRI images. The patient from the BMAC-MSCs group shows a local chondral lesion (indicated by the red arrow) on the patella. The green arrow highlights the observed effect of the therapy after one year.

The analysis showed that the groups did not differ from each other in the measurements and the final score in the BMAC-MSCs group was similar to that in the hUCB-MSCs group with no statistically significant differences. Example magnetic resonance images from the BMAC-MSCs group are shown below (Figure 4).

## 4. Discussion

The most important factor in any novel technique is safety. With BMAC-MSCs and hUCB-MSCs implantation, as with any relatively novel surgical treatment, there is an inherent risk of complications; nonetheless, this is another study [13], which has been conducted wherein no adverse effects were documented. During the whole follow-up period, there were no untoward events in either of the groups.

Furthermore, of equal importance, our approach has demonstrated comparable efficacy to the BMAC-MSCs group in terms of VAS, Lysholm, and KOOS assessments, despite a statistically significant difference in age between the two cohorts. Summarizing the obtained results, it can be stated that the subjective test outcomes in both groups improved over time after the surgery which can prove the efficacy of both of the methods. However, it is important to mention two significant differences between the groups—age and the size of the treated defect.

The statistical mean age difference between the two groups was nearly 10 years, with the patients in the hUCB-MSCs group being elder. Age assumes a substantial significance for two distinct reasons. Firstly, age has been identified as a significant factor impacting the clinical outcome of basic and widely known cartilage-repairing surgical procedures, i.e., microfractures, as evidenced by superior clinical outcome scores observed among younger patients (those below 40 years of age) [28]. Secondly, the abundance of stem cells and their differentiation capacity in bone marrow aspirate concentrate (BMAC) is commonly acknowledged to decline with age, leading to concerns regarding its applicability in older patients [29]. The differentiation, proliferative capacity, and metabolism profiles of MSCs are directly impacted by the age of their source. The younger the source, the greater the quantity of MSCs [30]. The improved outcomes observed in the elder group could potentially be attributed to the utilization of relatively young MSCs sourced from the human umbilical cord, in contrast to the BMAC cells that were of the same “age” as the patients. This distinction in cell age may have contributed to the enhanced results observed in the elder group. These findings can be partially explained by the proposed concept, which redefines MSCs as “Medicinal Signaling Cells” [31]. According to this paradigm, MSCs serve as dynamic and site-regulated entities within the body, acting as specialized “depots” that release trophic and immunomodulatory factors. Instead of primarily engaging in tissue formation, MSCs are posited to play a pivotal role in local injury responses, acting as crucial modulators of therapeutic processes.

There were also average lesion size differences in both groups. The mean lesion size in the BMAC-MSCs group was 336 mm^2^ versus 568 mm^2^ in the hUCB-MSCs group and the difference was statistically significant. As we mentioned before, well-established methods, i.e., OAT are considered to be the best method for larger size lesions [32] but only havethe potential to heal lesions smaller than 4.0 cm^2^ [8]. Taking into consideration the aforementioned factors and the size of the cartilage defects observed in the hUCB-MSCs group, our method shows potential for being effective in patients with large cartilage lesions that would not be amenable to treatment using conventional approaches used thus far.

Keeping in mind there are instances of biological and graft failures in any cartilage repair method, the majority of failures can be attributed to untreated underlying factors such as malalignment (56%), meniscal deficiency (19%), and instability (5%) [33]. Therefore, in our study we thoroughly recognized in protocol, addressed these background factors, and then during cartilage surgery added all the concomitant procedures as needed. It could partially explain good results in both groups.

Limitations of this study include the lack of full MRI data and not using a fully standardized and repeatable number of cells in each implantation, which is connected with the preparation method.

Another potential bias in our study arises from the utilization of different scaffolds. To the best of our knowledge, up to date, there are no studies comparing the direct impact of a specific matrix on treatment outcomes. The three matrices used in the study have different physical properties such as thickness, elasticity, adhesive properties, and mechanical strength, but their biological properties are very similar to each other [34].

Hyalofast^®^ (composed of hyaluronic acid) in our opinion has the best adhesive properties and is highly elastic, but it is susceptible to deformation and mechanical damage, making it best suited for small and deep defects. According to our perspective, Biomerg^®^ (composed of collagen layers) has the weakest adhesive properties and low elasticity, but its thickness and resistance to damage are its most significant advantages, making it suitable for covering large, deep defects in heavily loaded areas. However, its application is technically challenging for surgeons, so it is only used in exceptional cases. Chondrogide^®^ (composed of collagen I/III)occupies an intermediate position between the two aforementioned matrices and in our view is much more suitable for implantation compared to Biomerg or Hyalofast, hence, in the vast majority of cases, in a similar number in both groups, we used precisely this matrix. During surgical treatment, the surgeon selects the matrix best suited to the specific cartilage defect, considering three factors: the depth and size of the defect, the expected loads on the defect site due to its location in the joint, and the thickness of the surrounding healthy cartilage. Using only one type of matrix for all patients could significantly worsen the outcome of surgical treatment. According to the International Cartilage Repair Society, scaffolds should be biocompatible, causing little or no inflammatory response in the body, harmless when broken down by the body, porous enough to allow new cartilage growth and the eventual breakdown of the scaffold, while forming a “net” to maintain the most suitable environment for cartilage repair. Additionally, they should be easy to produce and versatile, depending on the size and shape required, and able to withstand the stresses and forces within the joint, such as the knee. According to both the manufacturer’s assurances and available research on individual matrices [35,36], the matrices we used fulfilled these criteria.

Due to the retrospective nature of this study, there was no predefined process for randomization in patient selection and treatment methods. As a result, this study may be susceptible to selection bias and performance bias, as the lack of randomization could have influenced the patient population and treatment outcomes. On the other hand, this is the first study that compares not commercially prepared products with hUCB-MSCs (Cartistem^®^) but individually prepared solutions of MSCs. Therefore, we believe that the somewhat limited sample size in both groups justifies the novelty of this study. We are aware that in the future, a multi-center randomized clinical trial with a larger number of patients would be necessary.

## 5. Conclusions

Our proposed method offers a safe, single-step, and satisfactory approach for managing full-thickness cartilage defects for the active population aged even over 50 years old. Nevertheless, a comprehensive, long-term prospective study with larger sample sizeand longer follow-up with second-look arthroscopy is warranted to thoroughly evaluate the therapeutic potential of this technique for both young and elderly patients with even large cartilage defects.

## Figures and Tables

**Table 1:** Demographic data of BMAC-MSCs and hUCB-MSCs groups.

	BMAC-MSCs (*n* = 19)	hUCB-MSCs (*n* = 20)	*p*-Value
Sex ^1^			
Female	7 (36.8%)	5 (25.0%)	0.432
Male	12 (63.2%)	15 (75.0%)	
Age	44.74 (14.65)	54.60 (9.02)	**0.018**
BMI	27.79 (4.26)	28.00 (3.15)	0.861
Lesion size (mm^2^)	336.00 (174.00)	568.00 (340.50)	**0.008**

^1^ For gender, frequencies and percentages were reported, for age, BMI (body mass index) and lesions, mean, and standard deviation were reported. **In bold statistically significant differences**.

**Table 2:** Differences over time in the context of VAS pain measurements within a group. Pre-surgery (pre), 6 M (6 M post-op), and 1 year after the surgery (1 Y).

Group	VAS	*M*	*SD*	*p*-Value	η^2^
BMAC-MSCs	1. pre	5.58	1.67	<0.001	
	2. 6 M	3.21	1.72		0.70
	3. 1 Y	2.16	1.89		
hUCB-MSCs	1. pre	5.60	1.93	<0.001	
	2. 6 M	3.00	1.26		0.76
	3. 1 Y	1.25	1.37		

Mauchly’s test results for the control group: χ^2^(2) = 1.95; *p* = 0.377; for the experimental group: χ^2^(2) = 8.55; *p* = 0.014. Significant differences were found between each measurement.

**Table 3:** Differences over time in the context of Lysholm scale within a group. Pre-surgery (pre), 6 M (6 M post-op), and 1 year after the surgery (1 Y).

Group	Lysholm Scale	*M*	*SD*	*p*-Value	η^2^_p_
BMAC-MSCs	1. pre	50.16	12.64	<0.001	
	2. 6 M	61.47	16.93		0.60
	3. 1 Y	75.37	18.34		
hUCB-MSCs	1. pre	56.20	9.76	<0.001	
	2. 6 M	65.85	11.37		0.74
	3. 1 Y	79.00	11.82		

**Table 4:** Differences over time in the context of KOOS scale within a group. Pre-surgery (pre), 6 M (6 M post-op), and 1 year after the surgery (1 Y). **In bold statistically significant differences**.

KOOS	BMAC-MSCs (*n* = 19)		hUCB-MSCs (*n* = 20)			
	*M*	*SD*	*M*	*SD*	*p*-Value	Cohen’s d
**Scale S**						
P0	53.63	14.96	56.80	12.09	0.471	0.23
6 M	67.00	15.19	68.15	14.36	0.809	0.08
1 Y ^a^	77.89	13.79	84.20	9.57	0.109	0.53
**Scale P**						
P0 ^a^	54.63	15.79	62.10	10.49	0.093	0.56
6 M ^a^	67.58	15.46	73.55	9.28	0.157	0.47
1 Y	73.11	16.10	82.00	12.39	0.060	0.62
**Scale A**						
P0	61.00	10.53	75.25	9.27	**<0.001**	1.44
6 M	74.11	13.96	77.25	14.16	0.489	0.22
1 Y ^a^	79.63	14.69	91.00	7.59	**0.006**	0.98
**Scale SP**						
P0	45.26	18.74	27.00	14.46	**0.002**	1.09
6 M	62.89	15.57	47.00	14.46	**0.002**	1.06
1 Y ^a^	69.74	18.22	69.25	10.67	0.920	0.03
**Scale Q**						
P0	46.89	16.14	36.70	9.93	**0.022**	0.77
6 M	62.05	14.43	55.70	17.43	0.224	0.40
1 Y	74.21	17.36	67.45	15.83	0.211	0.41

^a^—Annotation: Welch’s correction was applied.

**Table 5:** The outcome of MRI M-MOCART 2.0 Score.

MOCART	BMAC-MSCs		hUCB-MSCs			
	*M*	*SD*	*M*	*SD*	*p*-Value	Cohen’s d
1–6 M	45.71	13.99	40.32	14.21	0.351	0.38
≥6 M	47.05	11.85	36.39	14.26	0.077	0.82

## Data Availability

The data presented in this study are available on request from the corresponding author. The data are not publicly available due to privacy restrictions.
